# Use of Double Dose Intravenous Immunoglobulin Therapy in the Treatment of Streptococcal Toxic Shock Syndrome

**DOI:** 10.1155/crcc/9649482

**Published:** 2026-07-28

**Authors:** Yuning Xue, Alex Yartsev

**Affiliations:** ^1^ Westmead Intensive Care Service, Westmead Hospital, Westmead, Australia, nsw.gov.au; ^2^ Sydney Medical School, The University of Sydney, Sydney, Australia, sydney.edu.au

## Abstract

Streptococcal toxic shock syndrome (STSS) is a life‐threatening condition with increasing incidence and high mortality worldwide. It is characterised by hypotension and multiorgan failure, resultant of invasive group A Streptococcus (GAS) infections. Intravenous immunoglobulin (IVIG) has been proposed as an adjunctive therapy that reduces mortality in patients with STSS, and is thought to exert its effects by neutralising streptococcal superantigens and exotoxins to modulate the host immune response. Currently, there is a paucity of knowledge about the efficacy of repeated dosing if patients remain unstable after the first dose of IVIG. In this report, we describe the case of a woman in her 60s with STSS and meningoencephalitis, who demonstrated an improvement in haemodynamic status after the first dose of IVIG, but subsequently had rebound of hypotension and high vasopressor requirements, which resolved after a second dose of IVIG. This case highlights the potential need for repeated dosing of IVIG in STSS, in situations where an initial improvement in haemodynamic status is followed by deterioration.

## 1. Introduction

Streptococcal toxic shock syndrome (STSS) is a life‐threatening condition caused by invasive group A Streptococcus (GAS) infections and is characterised by hypotension (systolic blood pressure ≤ 90) and more than two of the signs of organ failure, including coagulopathy, renal impairment, liver involvement, erythematous rash, soft tissue necrosis and acute respiratory distress syndrome [1]. The incidence of invasive GAS disease, including STSS, in adults worldwide and in Australia has demonstrated variability across different regions and populations, but overall has shown an increasing trend. In Idaho, United States, the incidence of invasive GAS disease increased between 2008 and 2019 from 1.04 to 4.76 cases per 100,000 persons, and within these cases, the proportion of STSS rose from 0% to 6.4% during this period [2]. Similarly in Victoria, Australia, STSS accounted for 14.4% of invasive GAS disease, and the median annual incidence of invasive GAS increased from 3.1 to 5.2 cases per 100,000 persons between 2007 and 2017 [3, 4]. The mortality rate of STSS remains high at approximately 30% worldwide, and 23% in Victoria, Australia [3]. The incidence and mortality of invasive GAS disease are influenced by several risk factors, the most significant of which include intravenous drug use, indigenous status and haemodialysis patients. A retrospective review at a Melbourne tertiary centre revealed that 42% of patients with GAS bacteraemia had a history of intravenous drug use [5]. A disproportionately high incidence of invasive GAS disease was also found in Indigenous Australians, reportedly 69.7 per 100,000 in the Northern Territory compared with 8.8 per 100,000 in the non‐Indigenous population [6]. Haemodialysis patients have also been identified as a high‐risk group, with incidence of invasive GAS at 2205.9 per 100,000 persons in the Northern Territory [6].

Intravenous immunoglobulin (IVIG) has been supported by mechanistic data as an adjunctive therapy for STSS, and is thought to exert its immunomodulatory effects through two main mechanisms. Firstly, antibodies contained in IVIG neutralise streptococcal superantigens and exotoxins, which would have otherwise led to systemic inflammation and shock via the massive activation of T‐cells and release of proinflammatory cytokines such as IL‐6 and TNF‐*α* [7, 8]. Secondly, IVIG enhances the opsonisation of streptococci and facilitates phagocytosis [9].

The clinical efficacy of IVIG in STSS has been shown in existing literature both in international and Australian settings. A comparative observational study conducted in Canada found that 30‐day survival was 67% in patients with STSS and treated with IVIG, compared with 34% in those who did not receive IVIG [7]. In Australia, it has been demonstrated that in patients with severe invasive GAS infections, including STSS, necrotising fasciitis, septic shock and GAS cellulitis with shock, those treated with a combination of clindamycin and IVIG had significantly reduced mortality rates (7%) compared with those treated with clindamycin alone (15%), and those not treated with either therapy (39%) [10]. Based on existing evidence, the National Blood Authority in Australia developed two qualifying criteria for the use of IVIG in STSS, recommending it either for early use in those with probable or confirmed STSS, or in STSS ‘where rapid improvement is not obtained with fluid resuscitation, inotropes, surgery, antibiotic therapy and other supportive measures′ [11].

There is a paucity of evidence for the use of a double dose of IVIG in STSS. Variable IVIG dosing regimens between 0.2 and 3.6 g/kg have been used in prior literature, either as a single dose or divided over several days [7]. The current recommendation by the National Blood Authority in Australia is 2 g/kg as a single dose [11]. Repeated dosing in the event of ongoing clinical instability has also been described, but there is a paucity of research into its efficacy [7]. In this report, we describe a case of double dose IVIG used in the treatment of STSS, due to a rebound of shock after the first dose of IVIG, with subsequent improvement in haemodynamic status and vasopressor support requirements after the second dose.

## 2. Case Presentation

A non‐Indigenous woman in her 60s was brought to the emergency department by ambulance with fever, headache and a GCS of 12 (E4V3M5). Her past medical history included recent suppurative otitis media with perforated tympanic membrane, hypertension and anxiety (previously on venlafaxine and olanzapine). As per her husband, she had visited her grandson 8 days prior to presentation. At that time, her grandson was experiencing coryzal symptoms including cough, sore throat and rhinorrhoea. She had then developed left ear pain 7 days ago thought to be otitis media, but did not receive any antibiotics. She complained of generalised malaise, diarrhoea and a sore throat 2 days ago, then a frontal headache and fever 1 day ago. She was brought to the GP on the day of presentation due to deterioration in her condition—she became confused, demonstrated word‐finding difficulty and was speaking in inappropriate words, had reduced coordination, developed photophobia, became incontinent of urine, and vomited once. An ambulance was called, and her vital signs with the paramedics were as follows: temperature of 39.9°C, respiratory rate of 32 brpm, oxygen saturation of 92% on room air, heart rate of 130 bpm, systolic blood pressure of 142 mmHg and blood glucose level of 10.1 mmol/L. She was given ondansetron 4 mg for nausea/vomiting and fentanyl 50 mcg for pain.

On arrival to the emergency department, she remained febrile, tachycardic and tachypnoeic, and was hypertensive with a blood pressure of 185/99 mmHg. On examination, her airway was patent and her own. Bibasal crackles were heard on auscultation, and her heart sounds were dual and regular. She was diaphoretic and warm both centrally and peripherally. Her GCS was fluctuating between 14 and 15 (E4V4‐5M6), her pupils were equal at 3 mm and reactive to light, and she had no photophobia, neck stiffness or facial droop. Her power was intact in all four limbs, with normal tone and no evidence of clonus. Her cranial nerve examination was unremarkable. She demonstrated hyperreflexia on testing of the patellar reflex bilaterally, and her Babinski′s sign was equivocal on the right side, and down‐going on the left side. Her abdomen was soft and nontender with no signs of peritonism, and she was oliguric as measured with an indwelling catheter. Otoscopy of her left ear showed a perforated tympanic membrane with yellow exudate. Her COVID‐19 rapid antigen test was negative, and her urinalysis was negative for leukocytes and nitrite. Her chest x‐ray showed no obvious focal consolidations or effusions, and her ECG showed sinus tachycardia with widespread ST depression. Her venous blood gas showed respiratory alkalaemia, with a pH of 7.45, pCO2 of 24 mmHg, HCO3 of 17 mmol/L, base excess of −6.6 mmol/L, and a lactate of 9.3 mmol/L. Her blood biochemistry showed an acute kidney injury (AKI), with a creatinine of 143 *μ*mol/L, and a calculated eGFR of 33 mL/min/1.73 m^2^ (baseline creatinine 60 *μ*mol/L and baseline eGFR > 90 mL/min/1.73 m^2^). She had elevated C‐reactive protein to 215 mg/L and procalcitonin to 37.82 *μ*g/L, but low white cell count of 2.3 × 10^9^/L. Given the concerns for sepsis, she was given intravenous gentamicin 400 mg (5 mg/kg) and flucloxacillin 2 g as empirical treatment. She was also commenced on intravenous fluid resuscitation with crystalloids, with lactate down‐trending to 8.5 mmol/L after administration of 2 L of fluid.

Her CT brain demonstrated no acute intracranial pathology but found left mastoid and middle ear effusion in the context of known left otitis media and also noted an incidental 7‐mm right frontal meningioma. A lumbar puncture was performed in the emergency department under aseptic technique, and cloudy CSF was collected at an opening pressure of 22 cmH2O. She had a CSF glucose of 1.6 mmol/L, CSF protein of 4.81 g/L, total CSF leukocytes of 810 × 10^6^/L, total CSF erythrocytes of 135 × 10^6^/L, CSF polymorphs of 810 × 10^6^/L and no CSF mononuclears. Her CSF VDRL, fluorescent treponemal antibody absorption test and treponema pallidum particle agglutination test were nonreactive. Considering the likely diagnosis of meningoencephalitis, her empirical antimicrobial regimen was escalated to ceftriaxone 2 g, vancomycin 1 g, acyclovir 10 mg/kg TDS, and she was also given dexamethasone 8 mg.

Following the administration of dexamethasone, the patient vomited and subsequently aspirated on the vomitus. Her work of breathing increased, expiratory wheeze was heard in the right upper zone on auscultation, and her oxygen requirements escalated to 15 L/min high‐flow nasal prongs (50% FiO2) to maintain oxygen saturation over 90%. A repeat chest x‐ray showed consolidation in the bilateral lower lobes.

Six hours after arriving in the emergency department, she remained tachycardic, but increasingly hypotensive despite fluid resuscitation, with a blood pressure of 102/56 mmHg and MAP of 69. Her peripheries were cold and mottled. She was transferred to ICU and commenced on noradrenaline and argipressin, titrated to maintain MAP above 65. She was commenced on milrinone for disseminated intravascular coagulation (DIC), which was characterised by the presence of purpura fulminans, thrombocytopenia with a platelet count of 40 × 10^9^/L, prolonged PT (19 s), APTT (40 s) and INR (1.7), and low fibrinogen of 1.9 g/L. She was also intubated in ICU for worsening oxygen requirements of 70% FiO2 and 60 L/min flow.

Gram‐positive cocci were found on her CSF, blood culture and culture of her left ear exudate, soon confirmed to be *Streptococcus pyogenes* susceptible to penicillin and clindamycin. Her antibiotics regime was changed to clindamycin 900 mg TDS, benzylpenicillin 1.8 g 4‐h (renally adjusted) and ceftriaxone 2 g BD to cover for meningoencephalitis and aspiration pneumonia. She was continued on dexamethasone 12 mg BD. For her otitis media, she was commenced on 5 days of ciprofloxacin 0.3% ear drops, xylometazoline nasal spray and sodium chloride spray. For her kidney injury, she was commenced on continuous renal replacement therapy, but her benzylpenicillin dose remained unchanged as per the recommendations of Therapeutic Guidelines and Renal Drug Database [12, 13].

**Figure 1:**
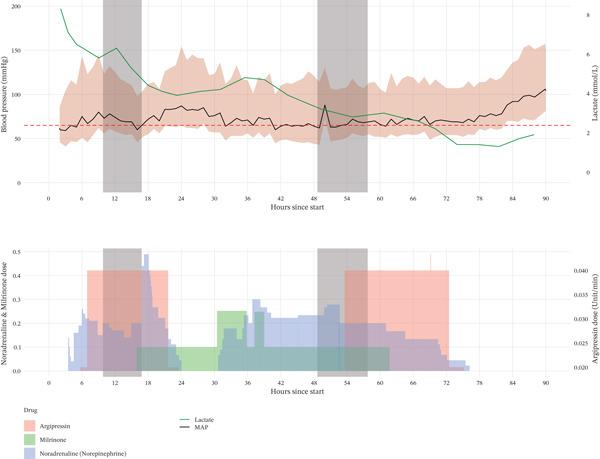
MAP, lactate and vasopressor requirements (argipressin, milrinone and noradrenaline) during the first 90 h of the patient′s ICU admission, with the timing of IVIG doses as marked by grey squares.

IVIG 200 g (2.5 g/kg) was given at this time (Day 1 in ICU), to which the patient showed a good response. Fourteen hours after the dose of IVIG (Day 2 in ICU), she was weaned off both noradrenaline and argipressin, and remained afebrile for the next 72 h. However, she was recommenced on noradrenaline 6 h later, and recommenced on argipressin the next day (Day 3 in ICU) as her MAP was unable to be maintained above 65. Her vasopressor requirements remained persistently high following the rebound of hypotension. A multidisciplinary discussion was held between the ICU team, the infectious diseases team and the immunology team on Day 3, in which all teams were in agreement that despite the lack of strong evidence for the use of a second dose of IVIG, its benefits outweighed the risks in this clinical scenario as the first dose showed an optimistic response, and that there were no other available treatment options to manage the patient′s deterioration. Following this discussion, a second dose of IVIG 200 g (2.5 g/kg) was given. The patient′s sedation was weaned to facilitate neurological assessment, and also to recruit her sympathetic response to assist in improving her haemodynamic status. Eight hours after the administration of the second dose of IVIG, the patient was weaned off noradrenaline and argipressin, with stable haemodynamic status and no further vasopressor requirements for the remainder of her hospitalisation. Figure 1 represents trends in MAP, lactate and vasopressor requirements during the first 90 h of the patient′s ICU admission, with the timing of IVIG doses as marked. She was stepped down from ICU after a 32‐day admission, and subsequently underwent amputations of her left lower leg, left 2nd–5th fingers and right forefoot due to gangrene secondary to sepsis and significant vasopressor requirements.

## 3. Discussion

Although existing literature acknowledges the use of IVIG in reducing mortality in patients with STSS, there is currently no strong evidence or guideline recommendations supporting the use of a double dose of IVIG for the treatment of STSS. In this report, we described the case of a patient with STSS, associated with meningoencephalitis, otitis media, DIC and AKI. She initially demonstrated a positive response to the first dose of IVIG, but subsequently had a rebound of hypotension and high vasopressor requirements, which resolved after a second dose of IVIG. This case highlights the potential need for repeated dosing of IVIG in STSS, in situations where an initial improvement in haemodynamic status is followed by deterioration.

The efficacy of IVIG as an adjunctive treatment for STSS has been demonstrated in existing literature as outlined above. In this case, the rebound of hypotension and vasopressor requirements may be attributed to the insufficiency of IVIG dose to counteract the ongoing *S. pyogenes* infection, as well as the superantigens and exotoxins it produces. Previous studies have used up to 3.6 g/kg in the management of STSS, compared with 2.5 g/kg used in this case [7]. Existing literature has also found significantly variable neutralising activity against GAS superantigens between different preparations of IVIG, or even different lots of the same preparation [8]. Therefore, higher or repeated doses of IVIG may be required to achieve the desired therapeutic effect, and this could explain the final resolution of the patient′s haemodynamic instability after the administration of two doses of IVIG. Further research into the optimal dosing strategy of IVIG in STSS can further elucidate the role of repeated dosing in light of evolving clinical status.

Although the patient′s rebound of haemodynamic instability resolved after the second dose of IVIG, we acknowledge that the temporal correlation does not necessarily imply causation. As the patient′s lactate level was already down‐trending prior to the second dose of IVIG, there is a possibility that the patient would recover from shock with standard treatment alone. However, this improvement in lactate level could be confounded by haemodilution from resuscitation with crystalloid and blood products, as the patient had a net 3.7L positive fluid balance at the time of administering the second dose of IVIG, meaning that the decrease in lactate concentration may not reflect improved tissue perfusion. Given the patient′s increasing vasopressor requirements during her rebound of haemodynamic instability, the treating team′s impression was that a second dose of IVIG should be given as an adjunctive therapy to manage the patient′s ongoing shock.

The initial use of empirical broad‐spectrum antibiotics was due to uncertainty about the causative agent, and the patient′s antibiotic regime had been continuously tailored as microbiological findings became available. Initial empirical treatment for sepsis of unknown origin with gentamicin and flucloxacillin provided adequate cover for *S*. *pyogenes*, the culprit organism for the patient′s STSS. Similarly, the change of antibiotics to target meningoencephalitis, ceftriaxone and vancomycin, also covered for *S. pyogenes*. After *S. pyogenes* was isolated on CSF, blood and otitis media exudate cultures, and was found to be susceptible to penicillin and clindamycin, treatment was adjusted a final time to ceftriaxone, benzylpenicillin and clindamycin, echoing current evidence of the reduction in STSS mortality with a combined treatment of clindamycin and IVIG [10].

On reflection of this case, it must be emphasised that close monitoring and timely intervention are vital in the management of STSS. Continuous monitoring of the patient′s haemodynamic status was achieved by her admission to ICU, and allowed for continuous assessment of her clinical trajectory, especially during periods of great change and uncertainty, namely the initial improvement after the first dose of IVIG was administered, followed by deterioration and rebound of shock and vasopressor requirements. This facilitated early decision‐making about the next steps in treatment, such that a multidisciplinary meeting was able to be organised in a timely manner, and the second dose of IVIG was given promptly, resulting in rapid improvement of the patient′s haemodynamic status.

## 4. Conclusion

IVIG is an adjunctive treatment for STSS and has been shown to reduce mortality in patients with this condition. For patients who demonstrated initial improvement in haemodynamic status after the first dose of IVIG but subsequently have rebound of shock and vasopressor requirements, a second dose of IVIG may be indicated.

## Funding

Open access publishing facilitated by The University of Sydney, as part of the Wiley ‐ The University of Sydney agreement via the Council of Australasian University Librarians.

## Consent

All the patients allowed personal data processing, and informed consent was obtained from all individual participants included in the study.

## Conflicts of Interest

The authors declare no conflicts of interest.

## Data Availability

The data that support the findings of this study are not available due to ethical or confidentiality restrictions.
